# Tuberculosis: A Silent Intruder in the Musculoskeletal Landscape of the Upper Extremity

**DOI:** 10.7759/cureus.69370

**Published:** 2024-09-13

**Authors:** Shobhana Rajasekar, Srinivasan Rajappa, Aravindhaa Ramasamy Giridharan

**Affiliations:** 1 Department of Hand Surgery, Sri Ramachandra Institute of Higher Education and Research, Chennai, IND; 2 Department of Orthopaedics, Sri Ramachandra Institute of Higher Education and Research, Chennai, IND

**Keywords:** anti-tubercular therapy, biopsy, musculoskeletal system, tuberculosis, upper limb

## Abstract

Tuberculosis (TB) is an infection that can occur in every organ of the body, including the musculoskeletal system. Musculoskeletal involvement in TB can be missed because of its non-specific clinical signs. The disease may mimic inflammatory arthritis, and high clinical suspicion is required when dealing with longstanding swelling of soft tissues, bones, or joints. This is a case series consisting of four patients diagnosed with TB of the musculoskeletal system of the upper extremity and treated with anti-TB drugs over a period of three years. The aim was to analyze the usual presentation pattern, time delay in diagnosis, the key diagnostic tool, and mainstay treatment of choice. All four patients were treated with a regimen of a combination of anti-TB drugs, initial splinting, and intensive physiotherapy for functional rehabilitation and had complete resolution of pain and infection. The operative treatment was usually limited and mainly included debridement along with biopsy for definitive diagnosis. The mainstay treatment had been appropriate drug therapy. Musculoskeletal TB can be treated effectively with anti-TB drugs. Confirmation of the diagnosis with biopsy is vital in prompt initiation of the appropriate treatment, which can lead to better outcomes in patients.

## Introduction

Tuberculosis (TB), primarily known as a respiratory infection caused by *Mycobacterium tuberculosis*, is an opportunistic infection that can manifest in various organs beyond the lungs. Among these, extrapulmonary TB, particularly affecting the musculoskeletal system, poses significant clinical challenges. Musculoskeletal involvement in TB can be missed because of its non-specific clinical signs [1]. The disease may mimic inflammatory arthritis, and high clinical suspicion is required when dealing with longstanding swelling of soft tissues, bones, or joints [2]. The upper extremities, including the skin, tendons, muscles, bones, and joints, can be sites of infection, leading to severe morbidity if not diagnosed and treated promptly [3].

Tuberculous infections in the upper extremities can present as soft tissue abscesses, arthritis, or osteomyelitis. Patients may experience persistent pain, swelling, and limited range of motion (ROM), which can significantly impact their daily activities and quality of life. Early recognition is crucial, as the symptoms may be insidious and easily mistaken for other musculoskeletal conditions.

The musculoskeletal system is involved in 1-3% of patients with TB and accounts for 30-40% of all extra-pulmonary TB [4,5]. The common sites are the spine (51%), pelvis (12%), hip and femur (10%), knee and tibia (10%), and ribs (7%) [5]. Tuberculous infection of the metacarpals, metatarsal, and phalanges of hands and feet is known as tubercular dactylitis [6]. The bones of the hand are affected more than the bones of the feet. In all, 85% of patients with tubercular dactylitis are younger than six years of age, and in children, multiple bones are involved [7]. Tubercular dactylitis in adults is rare, and commonly, a single bone is affected. Wrist joint TB is a rare complication of musculoskeletal TB and accounts for less than 1% of all osteoarticular TB. The reported incidence of elbow TB varies from 1% to 5% of all skeletal locations [8].

In this case series, we aim to summarize the current literature on the diagnosis and management of *M. tuberculosis* infection of the upper extremities. By synthesizing recent findings and guidelines, we seek to provide a comprehensive overview of the complexities involved in identifying and treating TB that affects the musculoskeletal system, particularly in the upper limbs.

## Case presentation

This is a case series consisting of four cases who were diagnosed with TB of the musculoskeletal system of the upper extremity and treated with anti-TB therapy (ATT) between 2021 and 2023 in the Department of Hand Surgery, Sri Ramachandra Institute of Higher Education and Research, Chennai, India. The aim was to analyze the usual presentation of patients with TB of the upper extremity clinically and radiologically, the time interval for diagnosis, the definitive diagnostic tool, and the mainstay treatment of choice. Retrospectively, data was collected from case sheets consisting of demographic data, history of presenting complaints including pain and swelling along with duration, pain score using visual analog scale (VAS), quick disabilities of the arm, shoulder, and hand (DASH) score, ROM of affected and appropriate joints, site of involvement, hematological parameters such as erythrocyte sedimentation rate (ESR) and C-reactive protein (CRP), radiological findings including X-radiation (X-ray) and magnetic resonance imaging (MRI), and histopathological examination (HPE) report.

The indications of surgery were limited, with debridement and biopsy being the most common procedures. All four patients, after confirmation of diagnosis, were treated with ATT as per the National Tuberculosis Elimination Program (NTEP) guidelines [9], along with splinting and physiotherapy for functional rehabilitation. Anti-TB chemotherapy of the initial intensive phase consisted of eight weeks (56 doses) of isoniazid (H), rifampicin (R), pyrazinamide (Z), and ethambutol (E), given in daily dosages, followed by a continuation phase consisting of 24 weeks of isoniazid, rifampicin, and ethambutol [9]. All the affected and appropriate joints were immobilized with a splint till the inflammation, swelling, and pain subsided. The period of immobilization ranged from 10 days to 21 days, depending on the needs of each patient, with a mean average of 2.2 weeks. This was followed by mobilization of the joint and strengthening exercises for two to six months. The mean follow-up period was 14.7 months. Follow-up data regarding the healing of the wound, hematological parameters such as ESR and CRP, pain score using VAS, quick DASH score, and ROM of the affected and appropriate joints of the last follow-up were recorded and evaluated. 

Study subjects

In our case series, four patients were included. Out of four patients, there were two male and two female patients. The right side was involved in two patients, and the left side in two patients. Age ranged from 22 to 68 years (mean age of 51.2 years). The average time to diagnose was seven months (range: six weeks to 12 months). 

Case 1

**Figure 1:**
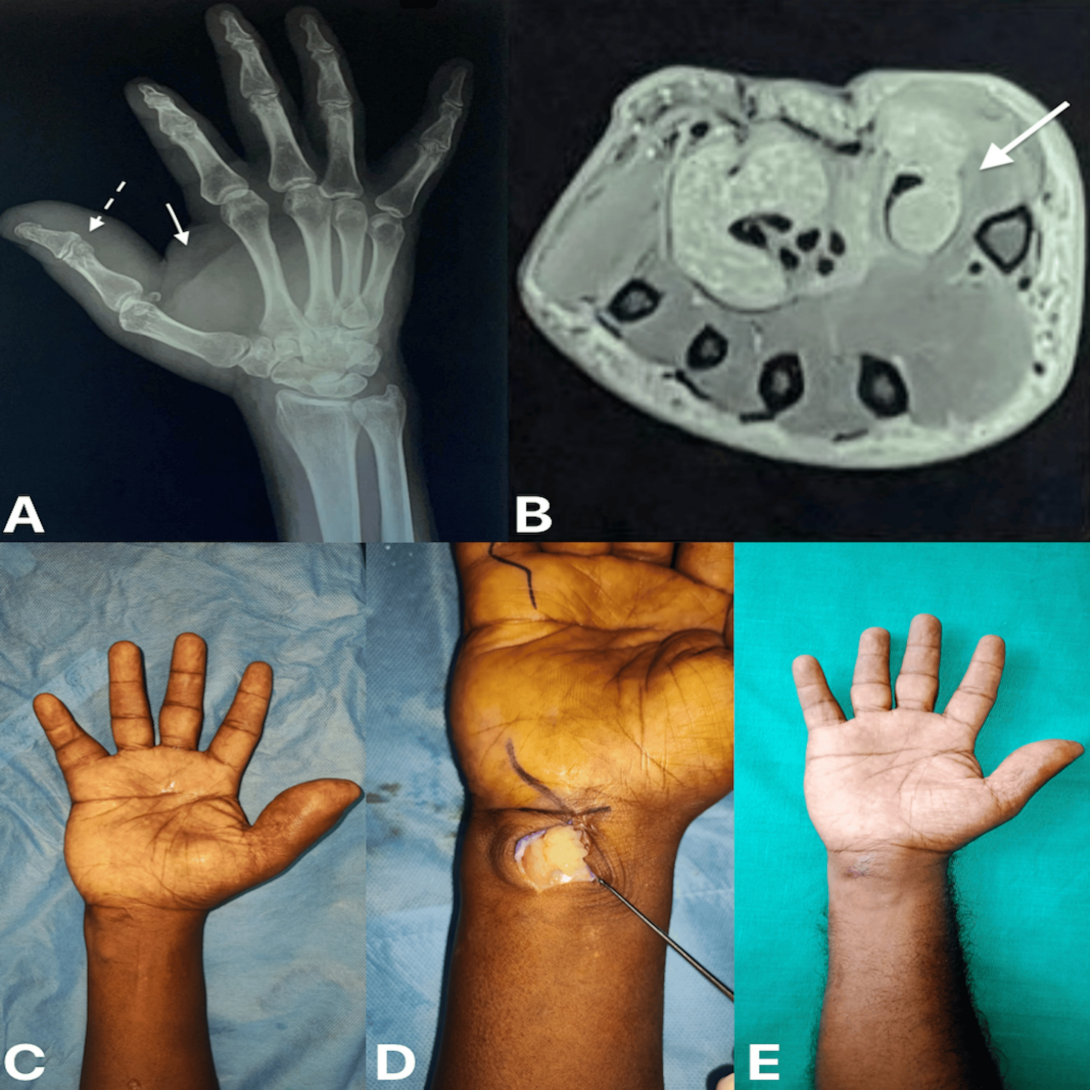
(A) Radiograph of the right hand with wrist: oblique view, (B) magnetic resonance imaging of right wrist: axial section, (C) pre-operative clinical picture of the right hand with wrist, (D) intra-operative picture showing rice bodies, and (E) one-year post-operative clinical picture of the right hand with wrist (A) The dotted arrow shows thumb interphalangeal joint destruction and erosion. The bold arrow shows a soft tissue shadow over the thumb and thenar region. (B) Arrow denotes synovial thickening in the palmar bursa.

A 56-year-old gentleman experienced pain and swelling in his right hand over the thumb extending into the thenar eminence and wrist (Figure 1C) for a year, with no history of fever, weight loss, or other symptoms. X-ray of the right hand with wrist showed a large soft tissue shadow over the right thumb and thenar region with destruction of the thumb interphalangeal joint and intra-articular erosions (Figure 1A). MRI showed fluid collection, subcutaneous edema involving the thumb, and moderate to severe synovial thickening in the palmar bursa extending from wrist level up to the metacarpophalangeal joint (Figure 1B). Intra-operatively, an incision was made over the wrist region, and the presence of multiple rice bodies was noted and removed (Figure 1D).

Table 1 summarizes the pre-operative findings of quick DASH score, VAS, and ROM of affected and appropriate joints at the time of presentation and the post-operative findings of quick DASH score, VAS, and ROM of affected and appropriate joints on the last follow-up of case 1.

**Table 1:** Pre-operative and post-operative quick DASH score, VAS, and ROM of affected and appropriate joints of case 1 DASH, disabilities of the arm, shoulder, and hand; VAS, visual analog scale; ROM, range of movements; IP, interphalangeal joint; MCP, metacarpophalangeal joint; “-” denotes empty space

Parameters	Pre-operative findings	Post-operative findings
Quick DASH score	66	33
VAS	8	3
ROM of affected and appropriate joints	-	-
Thumb IP	Nil	0-40°
First MCP	Full ROM, painful on extreme flexion	Full and pain-free
Wrist dorsiflexion	0-20°	0-40°
Wrist palmar flexion	0-10°	0-30°

Case 2

**Figure 2:**
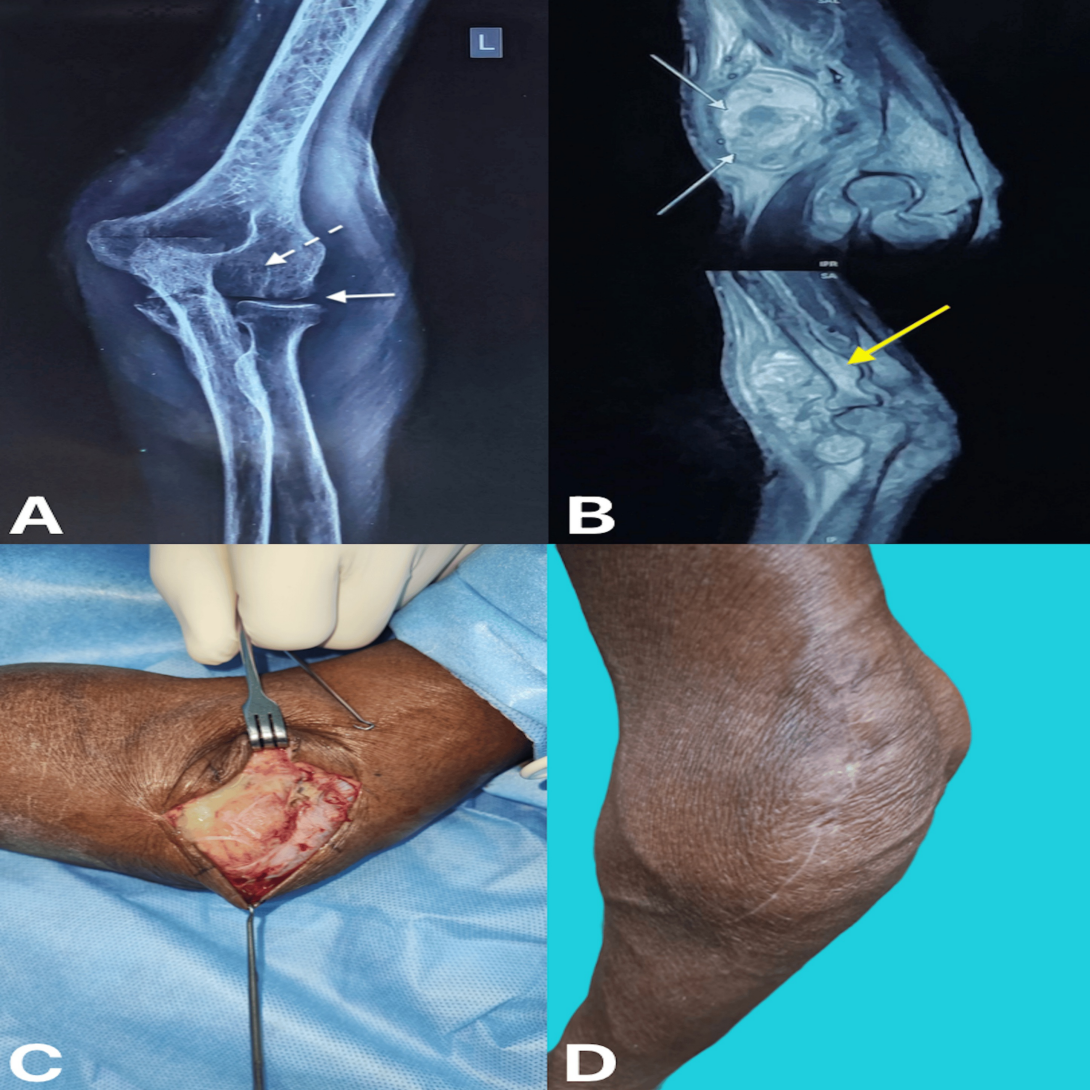
(A) Radiograph of anterior-posterior view of left elbow joint, (B) magnetic resonance imaging of left elbow showing sagittal section, (C) intra-operative picture showing infected synovium, and (D) four months post-operative clinical picture of left elbow (A) The bold arrow shows decreased joint space. The dotted arrow shows periarticular osteopenia. (B) The white arrow in the above image shows a soft tissue abscess. The yellow arrow in the image shows marrow edema.

A 68-year-old lady experienced pain and swelling over her left elbow for three months, with no history of fever. She had a history of weight loss for three months. X-rays showed arthritic changes, peri-articular osteoporosis, osseous erosions, and gradual narrowing of joint space (Figure 2A). MRI showed synovial thickening, erosions, joint effusion, marrow changes, and soft tissue abscess (Figure 2B). Intra-operatively, the abscess cavity was drained out, and infected synovium was noted, which could not be completely debrided (Figure 2C). The infected synovial tissue, which was debrided, was sent for biopsy.

Table 2 summarizes the pre-operative findings of quick DASH score, VAS, and ROM of affected and appropriate joints at the time of presentation and the post-operative findings of quick DASH score, VAS, and ROM of affected and appropriate joints on the last follow-up of case 2.

**Table 2:** Pre-operative and post-operative quick DASH score, VAS, and ROM of affected and appropriate joints of case 2 DASH, disabilities of the arm, shoulder, and hand; VAS, visual analog scale; ROM, range of movements; “-” denotes empty space

Parameters	Pre-operative findings	Post-operative findings
Quick DASH score	49.5	22
VAS	7	2
ROM of affected and appropriate joints	-	-
Elbow	40-110°	20-130°
Supination and pronation	Full and free	Full and free
Wrist dorsiflexion	Full	Full
Wrist palmar flexion	Full	Full

Case 3

**Figure 3:**
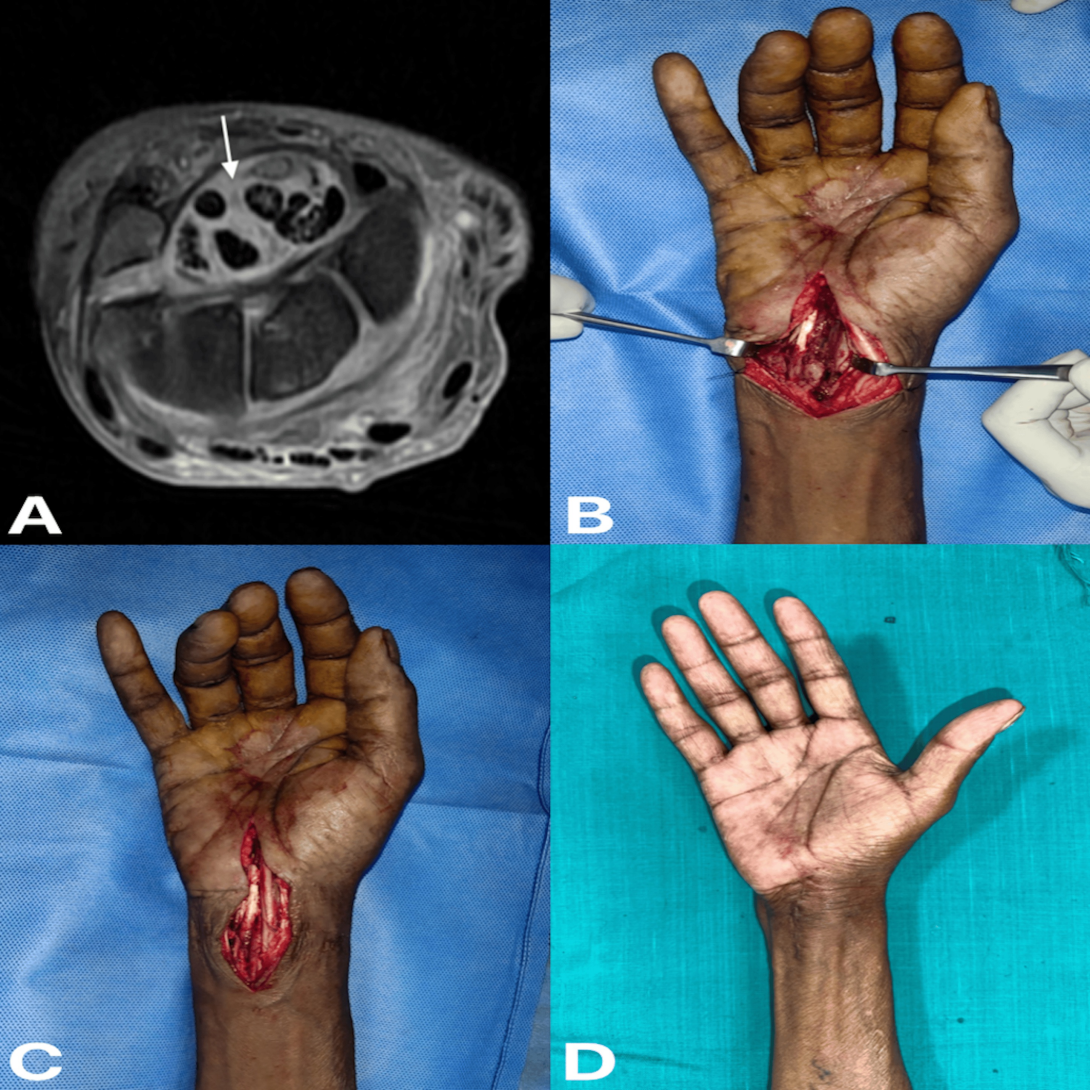
(A) Magnetic resonance imaging of right wrist showing axial section, (B) intra-operative picture showing infected synovium, (C) intra-operative picture showing median nerve decompression, and (D) six months post-operative clinical picture of right wrist (A) Arrow denotes synovial thickening and edema around the flexor tendon.

A 59-year-old gentleman experienced pain and swelling over his right wrist for six weeks, with no history of weight loss. He had a history of on-and-off fever for one month. X-rays showed juxta-articular osteopenia and well-aligned carpal bones with some soft tissue shadow. MRI showed significant edema over carpal bones and in the carpal tunnel, synovial thickening around the flexor tendons, and synovial fluid collection in the tendon sheath (Figure 3A). Intra-operatively, infected synovial tissue was present around the flexor tendons (Figure 3B), which was debrided, and median nerve decompression was done (Figure 3C).

Table 3 summarizes the pre-operative findings of quick DASH score, VAS, and ROM of affected and appropriate joints at the time of presentation and the post-operative findings of quick DASH score, VAS, and ROM of affected and appropriate joints on the last follow-up of case 3.

**Table 3:** Pre-operative and post-operative quick DASH score, VAS, and ROM of affected and appropriate joints of case 3 DASH, disabilities of the arm, shoulder, and hand; VAS, visual analog scale; ROM, range of movements; MCP, metacarpophalangeal joint; IP, interphalangeal joint; “-” denotes empty space

Parameters	Pre-operative findings	Post-operative findings
Quick DASH score	55	27.5
VAS	7	2
ROM of affected and appropriate joints	-	-
Wrist radial deviation	0-15°	0-15°
Wrist ulnar deviation	0-20°	0-30°
Wrist dorsiflexion	0-70°	0-70°
Wrist palmar flexion	0-60°	0-60°
MCP, IP of fingers and thumb	Reduced and stiff	Full and free ROM

Case 4

**Figure 4:**
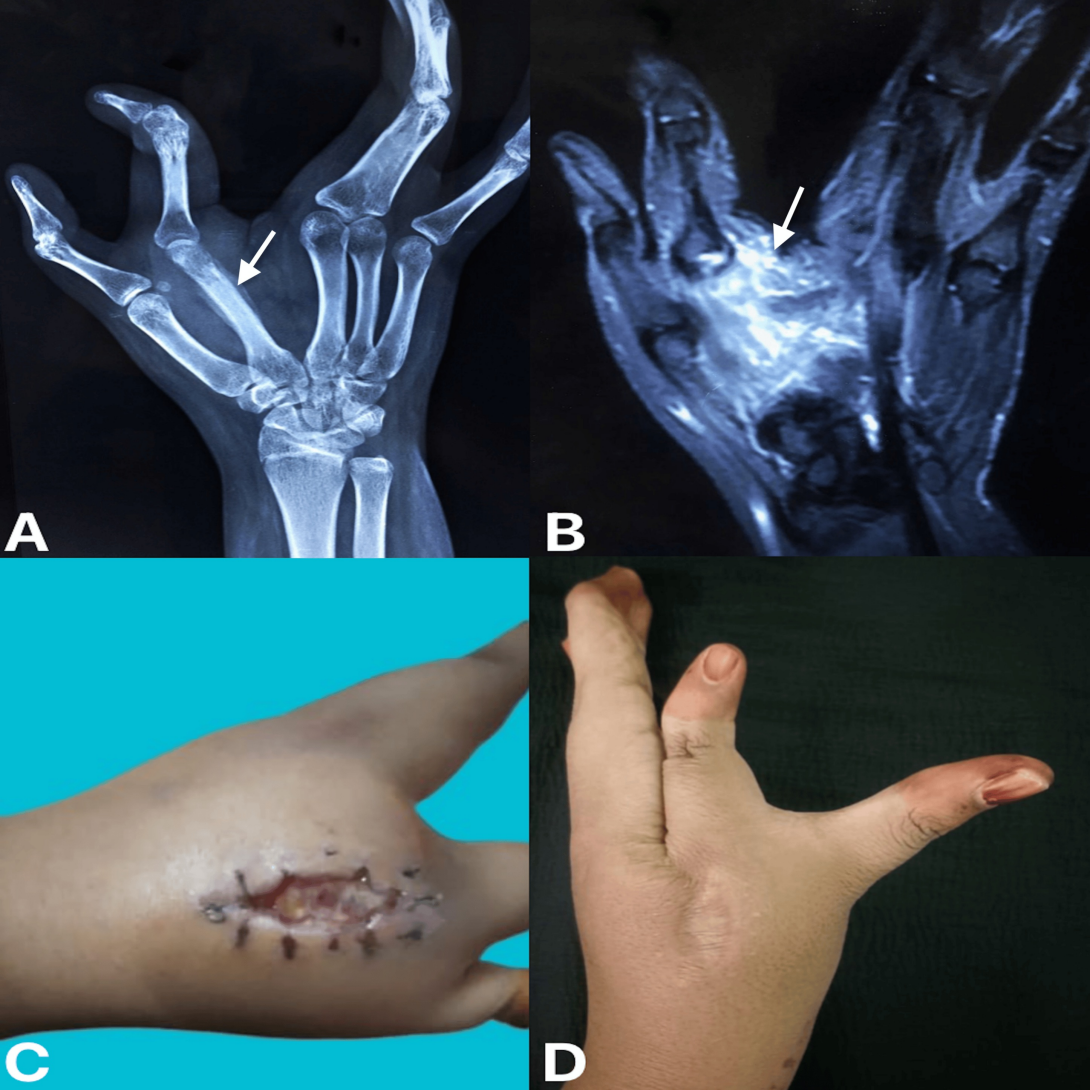
(A) Radiograph of left hand showing the anterior-posterior view, (B) magnetic resonance imaging of left hand showing coronal section, (C) four weeks post-operative wound showing sinus over the second metacarpal, and (D) 1.5 years post-operative clinical picture of left hand (A) Arrow shows rarefaction of the second metacarpal bone. (B) Arrow shows edema with fluid collection in the region of the second metacarpal bone.

A 22-year-old woman with a known case of left cleft hand experienced pain over her left hand in the region of the second metacarpal, along with swelling for 2.5 months. She had no history of fever or weight loss. X-ray showed rarefaction of the second metacarpal with periosteal reaction (Figure 4A), and MRI of the left hand revealed significant edema with the fluid collection and erosions over the neck and shaft of the second metacarpal bone (Figure 4B). Intra-operatively, an incision was made in the second metacarpal region, and the infected tissues were debrided and sent for biopsy. Post-operatively, the patient developed a sinus over the incision site (Figure 4C).

Table 4 summarizes the pre-operative findings of quick DASH score, VAS, and ROM of affected and appropriate joints at the time of presentation and the post-operative findings of quick DASH score, VAS, and ROM of affected and appropriate joints on the last follow-up of case 4.

**Table 4:** Pre-operative and post-operative quick DASH score, VAS, and ROM of affected and appropriate joints of case 4 DASH, disabilities of the arm, shoulder, and hand; VAS, visual analog scale; ROM, range of movements; MCP, metacarpophalangeal joint; IP, interphalangeal joint; “-” denotes empty space

Parameters	Pre-operative findings	Post-operative findings
Quick DASH score	71.5	41.25
VAS	8	2
ROM of affected and appropriate joints	-	-
Second MCP joint (index finger)	Restricted (0-45°) and pain on extreme flexion	Full and pain-free
Index finger IP joint	Full and free	Full and free
MCP, IP of other fingers and thumb	Full and free	Full and free
Wrist movements	Full and free	Full and free

For all four patients, the operative treatment was usually limited and included obtaining a biopsy and was sent to the pathology department, performing debridement (debridement of rice bodies), removal of an inflamed portion of the synovium in patients with tenosynovitis, incision, and drainage of abscesses and nerve decompression when indicated clinically. All four patients had consistent histopathology findings of well-defined epithelioid granulomas composed of epithelioid cells and giant cells of Langhans type surrounded by lymphocytes, along with caseating necrosis and granulation tissue formation. Cases 2 and 3 were reported to have acid-fast bacilli (AFB) positive, whereas cases 1 and 4 did not show AFB positive. Case 3 also had a positive GeneXpert report. 

The mainstay of treatment has been appropriate drug therapy. All four patients were treated with a regimen of a combination of ATT according to the NTEP guidelines, splinting, and intensive physiotherapy with functional rehabilitation whenever possible. Employing this regimen resulted in the resolution of infection and pain relief. The follow-up period of cases ranged from a minimum of eight months to a maximum of 24 months. Post-operatively, two patients (cases 2 and 4) had discharging sinus along the incision wound site, which resolved after two months of intense initiation phase of ATT. Hematological parameters like ESR and CRP were elevated at the time of presentation in all four cases, and there was a significant decrease in their post-operative values after the initiation of ATT.

Table 5 summarizes the pre-operative and post-operative ESR and CRP values of cases 1-4.

**Table 5:** Pre-operative and eight months post-operative ESR and CRP value of cases 1-4 ESR, erythrocyte sedimentation rate; CRP, C-reactive protein

Case number	ESR (reference range: 4-12 mm/h)		CRP (reference range: 0-0.8 mg/dL)	
	Pre-operative value	Post-operative value	Pre-operative value	Post-operative value
Case 1	60	6	8	0.4
Case 2	48	5	9.5	0.6
Case 3	37	11	7.5	0.7
Case 4	36	4	6	0.3

For all four patients, there was a significant decrease in both quick DASH score and VAS, with quick DASH score pre-operatively and post-operatively averaging 60.5 and 30.9, respectively, and VAS pre-operatively and post-operatively averaging 7.5 and 2.3, respectively. In our case series, we observed that there was a fair amount of improvement in ROM following the treatment. In case 3, the patient mainly had pain and swelling of the wrist. There was not much restriction of movements at the wrist, but there was restriction of movements in the fingers and thumb at the IP and MCP joints. Almost one month after the initiation of treatment, pain and infection completely resolved. As far as ROM was concerned at the wrist, there was no difference, but almost complete ROM was regained at the IP and MCP joints of the fingers and thumb. The follow-up radiograph of all four patients showed signs of healing and resolution of infection.

## Discussion

Extra-pulmonary TB occurs as a result of liquifying granuloma getting eroded into blood vessels, resulting in blood-borne spread of the bacilli, with seeding in the sites outside the lungs [10]. TB infection of the musculoskeletal system starts as synovitis, causing joint effusion and erosions, leading to the involvement of para-articular soft tissue [5]. This involvement may be confined to muscle or rarely sub-cutaneous tissue. When left untreated, it leads to marked juxta-articular bone demineralization and local bone destruction. According to Abidin, hand involvement is seen in 10% of patients with musculoskeletal TB, and the dominant hand is affected commonly [10]. They have also stated that local findings at the time of surgical exploration include granuloma, areas of fibrosis, and classical rice bodies. Rice bodies were first described in 1895, and they represent intra-synovial masses resembling rice grains [11].

Clinically, musculoskeletal TB presents with a gradual onset of pain, swelling, decrease in ROM, and deformity. The common diagnostic confusion in musculoskeletal TB is due to the similarity of the disease with the more common pathologies like inflammatory arthritis, rheumatoid arthritis, low virulence pyogenic arthritis, gout, and sometimes neoplasm. So, high clinical suspicion is required when dealing with long-standing soft tissue swelling and swelling of bone and joints, along with pain. 

Even though, in many cases, a biopsy or maybe a culture specimen is forced to give a conclusive analysis, it is important that the radiologist and clinicians have an understanding of the typical distribution, pattern of presentation, and imaging manifestation of musculoskeletal TB [5]. Radiological X-ray features of musculoskeletal TB are non-specific and can often delay diagnosis but may include bone marrow edema, joint space narrowing, osteoporosis, osseous erosions, or lytic lesions [12]. The surrounding tissue may show synovitis, joint effusions, tenosynovitis, soft tissue collections, or myositis [6]. MRI is a more specific investigation because of excellent soft tissue demarcation. T2-weighted images give an outline of fluid content inside the synovium with a mixed solid cystic appearance and thickening of the synovial membrane. It may also show increased vascularity, reactive inflammation, and swelling around the tendon [10]. To reach a definitive diagnosis, a tissue biopsy should be taken for microscopy, culture, and histology [13]. Tuberculous bacilli are often not seen in Ziehl-Neelsen staining or grown in culture, and the diagnosis often has to be made based on the granulomatous appearance histologically, along with high clinical and radiographic suspicion [14]. Along with conventional tests for the detection of *M. tuberculosis*, rapid molecular tests like GeneXpert MTB are also available. GeneXpert MTB is the only rapid molecular test recommended by the WHO for the rapid diagnosis of TB. Extrapulmonary samples reveal the high sensitivity and specificity of GeneXpert for the diagnosis of extrapulmonary TB as compared to those found for pulmonary specimens [15,16].

There are no clear recommendations regarding which cases can be managed medically and which would require surgical intervention. Keeping this in mind, our evaluation of cases, although retrospective, showed that early surgical intervention of debridement along with biopsy for confirmation of diagnosis and prompt initiation of anti-TB chemotherapy treatment resulted in halting the disease progress with complete resolution of pain and inflammation.

Previous authors like Dhillon et al. have reported that where surgical intervention of debridement had been done, even though some cases did not show clinical and radiological improvement after four to six weeks of anti-TB chemotherapy, several patients benefited as there was rapid local control of disease process, along with improvement in ROM [17]. They have also mentioned that surgical intervention could appropriately alter the outcome, especially in patients with extra-articular involvement close to the joint. It helps to avoid complications such as spontaneous tendon rupture and possible functional limitations. We also analyzed from our case series that excision of granulation tissue is important; though it may not be completely possible, it helps to debulk the disease load [10]. All the initial surgical procedures consisted of debridement +/− synovectomy and biopsy, which helped confirm the diagnosis and promptly start on appropriate ATT.

Cases presenting with pure synovial involvement usually respond well to anti-TB chemotherapy, short-term immobilization, and adequate physiotherapy. The ROM of relevant joints improved probably because muscle spasms subsided with reduced disease activity. Studies have suggested surgical debridement with excision of involved synovium, followed by treatment with an anti-TB medication regimen, as the treatment of choice [18].

In our series, cases 1 and 2 did not have complete gain of ROM of the involved joints but had fair improvement in ROM with painless and stable joints. This has been the experience of many previous authors [5,14].

Chen et al. [19] mentioned in their article that even patients with advanced disease with joint involvement, synovectomy, and intra-articular debridement, along with continuous passive movements in the post-operative period, gained a good ROM. 

We also observe that the need for surgical intervention might not be a true representation of the natural history of early diagnosed musculoskeletal TB; it reports a situation that has been reached due to diagnostic delay. So, in our series, we emphasize that timely diagnosis is most important in halting the progress of disease. Retrospectively, we tried to correlate the clinical and radiological features, delay in presentation, and the type of therapeutic measures undertaken with the functional end result [17]. 

Medical management is still the backbone of the treatment of TB infection. According to Tacata and Orillaza [20], the need for surgery may be a sign of a deeper, more difficult problem, as it is usually indicated for cases of synovitis, arthritis, or severe infection. They also reported that in a few patients, even after treatment, some of the disability persists. It may be due to the fact that TB can cause significant destruction of bone and soft tissue, especially because of its chronic course and often tolerable initial symptoms.

This again suggests that early diagnosis and timely start of treatment with ATT are key for the complete resolution of musculoskeletal TB without residual deformity. In our case series, there was complete resolution of pain and infection. There was no recurrence of infection, and there was no need for any further surgical intervention after the initiation and completion of ATT.

This case series had a few limitations. First, it was a retrospective study, and second, it was a small sample size. This didn’t achieve adequate power for statistical analysis. A prospective analytical study can yield more concrete conclusions.

## Conclusions

Musculoskeletal TB can be treated effectively with the regimen of a combination of ATT, initial splinting, and intensive physiotherapy for functional rehabilitation whenever possible. The initial surgical procedure consisting of debridement, with or without synovectomy, along with biopsy, helped confirm the diagnosis and promptly start on appropriate treatment with ATT. This paved the path to halt the progress of disease, reduce the disease load, and control the residual deformity, leading to a better outcome in patients. Early diagnosis and management are key to successful treatment.
